# Antidiabetic activity of isoquercetin in diabetic KK -A^y ^mice

**DOI:** 10.1186/1743-7075-8-85

**Published:** 2011-12-02

**Authors:** Rui Zhang, Yang Yao, Yingping Wang, Guixing Ren

**Affiliations:** 1 Institute of Crop Science, Chinese Academy of Agricultural Sciences, South Xueyuan Road, Haidian District No.80, 100081 Beijing, People's Republic of China

**Keywords:** isoquercetin, blood glucose, antidiabetic activity, KK -A^y ^mice

## Abstract

**Background:**

Tartary buckwheat bran is an important natural source of quercetin and isoquercetin. Quercetin and isoquercetin are both powerful α-glucosidase inhibitors. Although the IC_50 _of isoquercetin as α-glucosidase inhibitor was much higher than that of quercetin, the bioavailability of isoquercetin was higher than that of quercetin. Hence, we are interested in the antidiabetic effect of isoquercetin in diabetic KK -A^y ^mice.

**Methods:**

The hypoglycemic effect of isoquercetin in a type 2 diabetic animal model (KK-A^y ^mice) was studied. Isoquercetin was administrated at doses of 50, 100 and 200 mg/kg for 35 days.

**Results:**

It was found that fasting blood glucose concentration was decreased with the 200 mg/kg group (*p *< 0.01) the most efficient compared with the diabetic control group. In addition, there was significant decrease in plasma C-peptide, triglyceride, total cholesterol and blood urea nitrogen levels after 35 days. Meanwhile, glucose tolerance was improved, and the immunoreactive of pancreatic islets β-cells was promoted.

**Conclusions:**

These results suggest that isoquercetin had a regulative role in blood glucose level and lipids, and improved the function of pancreatic islets. Isoquercetin may be useful in the treatment of type 2 diabetes mellitus.

## Introduction

Diabetes, especially type 2 diabetes, is rapidly becoming an enormous health burden by decreasing quality of life and causing death and disability in the whole world, all at a huge economic cost. Diabetes is, in part, related to the amount of carbohydrates in the diet. Acting as a key enzyme for carbohydrate digestion, intestinal α-glucosidase is one of the glucosidases located at the epithelium of the small intestine. α-Glucosidase has been recognized as a therapeutic target for modulation of postprandial hyperglycemia, which is the earliest metabolic abnormality to occur in type 2 diabetes mellitus [1,2]. The inhibition on intestinal α-glucosidases would delay the digestion and absorption of carbohydrates and consequently suppress the postprandial hyperglycemia [3,4].

Tartary buckwheat bran is an important natural source of quercetin and isoquercetin. Quercetin and isoquercetin are both powerful α-glucosidase inhibitors [5]. The IC50 of quercetin was about five times lower than that of acarbose which has been used as effective α-glucosidase inhibitors to delay glucose absorption in clinical [6]. Quercetin significantly decreased the plasma glucose level and improved the biochemical profiles in the streptozocin-induced diabetic rats in a dose-dependent manner [7].

Diabetic KK-A^y ^mice have been frequently used as an animal model for noninsulin-dependent diabetes [8,9]. The symptoms of this animal model are similar to diabetic patients and the mice exhibit metabolism abnormalities such as an absolute or relative lack of insulin, hyperglycemia and glucose intolerance, higher lipid and so on. We are interested in the antidiabetic effect of isoquercetin in KK -A^y ^mice. Although the IC_50 _of isoquercetin as α-glucosidase inhibitor was much higher than that of quercetin [5], the bioavailability of isoquercetin was higher than that of quercetin [10]. Morand et al. [11] also demonstrated that isoquercetin was better absorbed than quercetin by rats. Furthermore, isoquercetin has been shown to play protective roles against lipid peroxidation and oxidative stress [12]. Accumulated evidence has suggested that diabetic patients are under oxidative stress with an imbalance between the free radical-generating and radical-scavenging capacity. The increased free radical production and reduced antioxidant defense may partially mediate the initiation and progression of diabetes-associated complications [13,14]. Here we reported our investigations on the effect of isoquercetin on diabetic KK-A^y ^mice.

## Materials and methods

### Preparation of isoquercetin from Tartary Buckwheat

The isoquercetin was prepared from tartary buckwheat provided by the Chinese National Genebank (Beijing, China) using the method of Chang and Muir [15]. Briefly, buckwheat was extracted with 50% methanol aqueous solution at 40°C for 3 hours. After vacuum filtration at 50°C, the supernatants were combined and concentrated to one-third of the volume under a reduced pressure using a rotary evaporator. The concentrate was dispersed in 500 ml of water (solid:liquid ratio = 1:100). Then the dispersion was heated to 80°C and the pH adjusted to 4, followed by the addition of naringin enzyme. After further purification by a preparative HPLC method, the isoquercetin purity was 96.7%, which was detected by an analytical HPLC procedure.

### Design of animal experiment

Experiments were carried out according to the method of Yao et al. [16]. Forty male diabetic KK-A^y ^mice [17] were obtained from the Department of Laboratory Animal Science Center (Beijing, China). The diabetic mice were divided into four groups according to their weights and blood glucose levels to make the average weights and blood glucose levels similar among the groups: group I, control diabetic animals (n = 10); group II, diabetic animals given 200 mg of isoquercetin/kg, n = 10); group III, diabetic animals given 100 mg of isoquercetin/kg, n = 10); group IV, diabetic animals given 50 mg of isoquercetin/kg, n = 10). Body weights were measured during the treatment. All mice were housed individually in stainless steel wire-bottom cages in an air-conditioned room kept at controlled ambient temperature (22 ± 1°C), humidity (50 ± 10%), and a 12 h light/dark cycle. The composition of the diets fed to the mice was shown in Table 1. They were allowed free access to the diet and water. The level of food intake was measured weekly. The experiment was carried out according to the European Community Guidelines for the Use of Experimental Animals and approved by the Peking University Committee on Animal Care and Use.

**Table 1:** Composition of the Experimental Diet

Content	KK-A^y^
corn starch	28.21
soybean meal (%)	11.62
soybean powder (%)	4.13
wheat flour (%)	14.00
wheat bran (%)	7.42
fish powder (%)	0.84
CaCO_3 _(%)	1.33
CaPO_4 _(%)	1.54
mineral (%)	0.56
vitamin (%)	0.07
soybean (%)	0.28
sucrose (%)	10.00
lard (%)	10.00
Yolk powder (%)	10.00

### Fasting blood glucose levels and oral glucose tolerance test (OGTT)

Blood samples were taken from the tail vein weekly after overnight fasting. Glucose was determined according to the method of Yao et al. [16] using glucose analyzes (ACCU-CHEK Active, Roche, Shanghai, China). On the morning of OGTT, fasting animals were given glucose orally (2 g/kg). Blood glucose levels were measured at 0 (before oral glucose), 30, 60, and 120 min after glucose administration.

### Plasma biomarker analyses

Blood was collected from abdominal artery into a heparin-coated tube and centrifuged at 1000 g for 15 min at 4°C, the plasma was colleted and stored at -20°C until analysis [18]. Plasma insulin (DSL-1600 Insulin RIA kit, Diagnostic Systems Laboratories, USA) level was measured based on a radio-immunometric assay. C-peptide (ADL, San Diego, CA) and glucagon (RapidBio Laboratory, Calabasas, CA) were determined using commercial enzyme-linked immunosorbent assay kits. Plasma total cholesterol, triglycerides, and blood urea nitrogen (BUN) were measured using an autobiochemical analyzer (Hitachi 7600, Japan) [19-21].

### Immunohistochemical evaluation on pancreas

The pancreas was removed immediately after sacrifice and rinsed in ice-cold saline. The tissue samples were fixed in paraformalclehyde, dehydrated in a graded series of ethanol, and embedded in paraffin wax before sectioning. Sections were immersed in a solution of 3% H_2_O_2 _for 10 min then preincubated with nonimmune serum for 15 min and subsequently replaced with the mouse anti-insulin antibody (1:200, SP-9000, ZYMED, CA) for incubation at 4°C for 16 h. Biotinylated goat anti-mouse immunoglobulin was used as a secondary antibody. They were labeled with streptavidin peroxidase followed by incubation with the secondary antibody at 37°C for 30 min. The localization of the antigen was indicated by a brown color obtained with 3-amino-9-ethyl-carbazole (AEC) as a chromogenic substrate for peroxidase activity. Slides were counterstained with hematoxylin for microscopic observation. The specificity of the immunohistochemical staining was checked by omission of the primary antibody or by using an inappropriate antibody (antigastrin). More than 10 islets in each mice group were randomly selected and transferred to a pathology image analyzing system. Staining signals of the islets selected on the captured image were converted to gray density which can be automatically calculated as a staining intensity per unit area.

### Statistical analysis

All values were expressed as mean ± SD. Data were analyzed using one-way analysis of variance (ANOVA). Differences with *p *< 0.05 were considered to be significant.

## Results and discussion

### Food intake and body weight

KK-A^y ^mice have high fasting and nonfasting blood glucose levels similar to human type 2 diabetes. The present study found that isoquercetin had no effect on the final body weight of KK-A^y ^mice during the 5-week period of treatment (Table 2).

**Table 2:** Effect of Isoquercetin on Body Weight and Food Intake (week) in Diabetic KK-A^y ^

	Initial body wt (g)	Final body wt (g)	Food intake (g)
control diabetic mice	36.49 ± 1.72	37.51 ± 1.42	11.56 ± 0.97
diabetic mice given 200 mg of isoquercetin/kg	35.43 ± 1.35	36.86 ± 1.87	12.32 ± 1.19
diabetic mice given 100 mg of isoquercetin/kg	37.21 ± 1.79	37.75 ± 1.68	10.08 ± 0.95
diabetic mice given 50 mg of isoquercetin/kg	37.09 ± 1.40	37.93 ± 1.53	11.53 ± 1.62

### Fasting blood glucose levels and oral glucose tolerance

**Figure 1:**
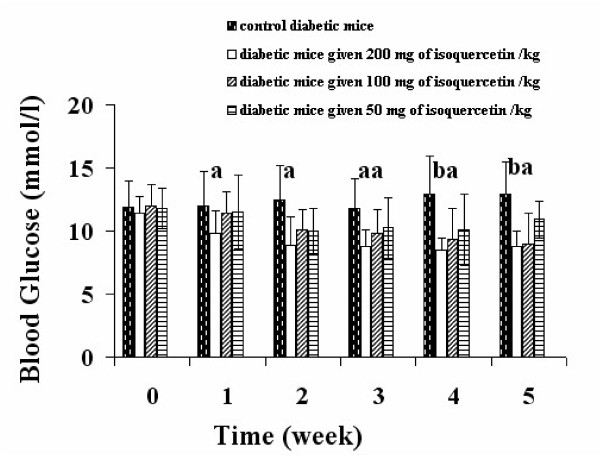
**Changes in blood glucose levels in different experimental groups**. Each column represents the mean ± S.D. of 10 animals. ^*a *^P < 0.05, *^b^*P < 0.01, *^c^*P < 0.001, compared with the control.

**Figure 2:**
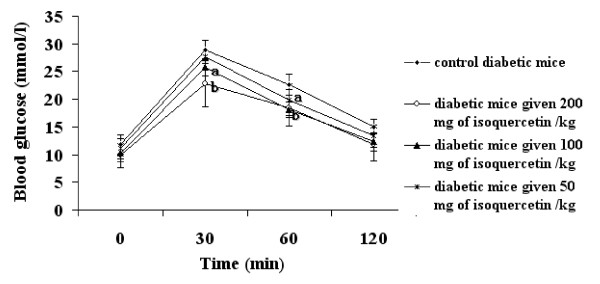
**Oral glucose tolerance test at the end of the 5-week treatment**. *^a^p *< 0.05, *^b^p *< 0.01 compared with the control.

Isoquercetin was hypoglycemic in KK-A^y ^mice (Figure 1). Oral administration of isoquercetin for 5 weeks caused a dose dependent decerease in blood glucose level compared with the control diabetic group (Figure 1). Isoquercetin appeared to improve the glucose tolerance in KK-A^y ^mice (Figure 2). The KK-A^y ^control mice showed a sharply increased blood glucose concentration at 30 min after glucose loading and maintained this high level for over an additional 60 min. All isoquercetin-treated KK-A^y ^mice showed decreases in blood glucose levels at 60 and 120 min compared with the control KK-A^y ^mice (Figure 2). Isoquercetin is a potential substrate for the lactase phloridzin hydrolase in the brush border membrane [22-24]. It is assumed that the hydrophilic monoglucoside might concentrate at the brush border membrane and be deglycosylated by this enzyme. The lipophilic aglycone is thought to be able to penetrate the apical enterocyte membrane by passive diffusion. Consequently, the higher concentration of quercetin originating adjacent to the apical membrane would result in a higher diffusion rate at this site and, thus, in greater absorption of the isoquercetin.

### Plasma biomarker levels

Isoquercetin (200 mg/kg) significantly lowered the levels of plasma insulin and C-peptide (*p *< 0.01), which is a byproduct of insulin production. Newly C-peptide levels are measured instead of insulin levels because insulin concentration in the portal vein ranges from two to ten times higher than in the peripheral circulation. The liver extracts about half the insulin reaching it in the plasma, but this varies with the nutritional state. The pancreas of patients with type 1 diabetes is unable to produce insulin and therefore they will usually have a decreased level of C-peptide, whereas C-peptide levels in type 2 patients are normal or higher than normal. Measuring C-peptide in patients injecting insulin can help to determine how much of their own natural insulin these patients are still producing.

In this study, the KK-A^y ^mice displayed hyperglucagonemia (*p *< 0.001) and isoquercetin lowered the plasma glucagon level compared to the control diabetic mice (Table 3). Glucagon is one of several hormones that possess antagonistic action against insulin that can exacerbate the metabolic consequences of insulin deficiency. The suppression of endogenous glucose production has been reported to be not just as a simple response to insulin but rather as a complex interplay between the action of glucagons and insulin [25]. Thus, the improvement in hyperglycemia by isoquercetin could be partly attributable to the amelioration of hyperglucagonemia.

**Table 3:** Plasma Parameters in Diabetic KK-A^y a^

	C-peptide (ng/mL)	glucagons (pg/mL)	triglycerides (mmol/L)	total cholesterol (mmol/L)	BUN ^b^ (mmol/L)
control diabetic mice	0.84 ± 0.09	107.73 ± 4.65	1.03 ± 0.12	4.98 ± 0.63	11.79 ± 2.40
diabetic mice given 200 mg of isoquercetin/kg	0.65 ± 0.21 a	67.29 ± 6.37c	0.83 ± 0.14 b	3.79 ± 0.67 a	9.35 ± 0.49 a
diabetic mice given 100 mg of isoquercetin/kg	0.76 ± 0.13	72.81 ± 7.58 b	0.86 ± 0.09 a	4.30 ± 0.36	11.26 ± 0.81
diabetic mice given 50 mg of isoquercetin/kg	0.81 ± 0.26	103.52 ± 8.29	0.90 ± 0.11 a	4.74 ± 0.52	10.94 ± 1.38

^a ^"a", *P *< 0.05; "b", *P *< 0.01; "c", *P *< 0.001, compared with the diabetic control mice. ^b ^BUN, blood urea nitrogen.

The most common lipid abnormalities in diabetes are hypertriglyceridemia and hypercholesterolemia [26]. In the present study, plasma lipids including cholesterol and triglycerides in diabetic mice were elevated (Table 3). Isoquercetin (200 mg/kg) decreased the level of triglyceride and total cholesterol may be due to the increased insulin releasing capacity [27].

Diabetic hyperglycemia induces the elevation of plasma urea nitrogen, which is considered to be a marker of renal dysfunction [28]. As shown in Table 3, plasma urea in isoquercetin group (200 mg/kg) reduced significantly plasma urea nitrogen by 12.8% (*p *< 0.05) compared with the value of the diabetic control group, indicating that isoquercetin may capable of ameliorating the impaired diabetic kidney function in addition to its hypoglycemic control.

### Immunohistochemical level on pancreas

**Figure 3:**
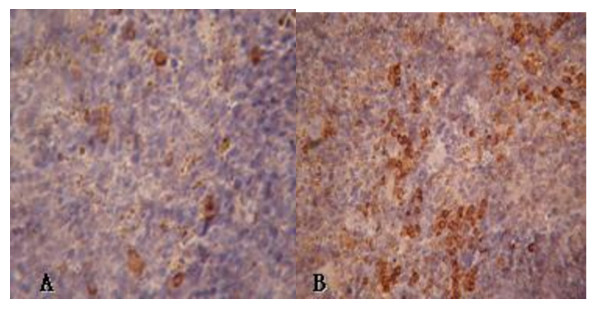
**Immunohistochemical evaluation on pancreas (400 ×): (A) Diabetic KK-A^y ^mice showing the decrease of insulin immunoreactivity, (B) Diabetic KK-A^y ^mice orally given isoquercetin (200 mg/kg) showing an improvement in insulin immunoreactivity**.

**Figure 4:**
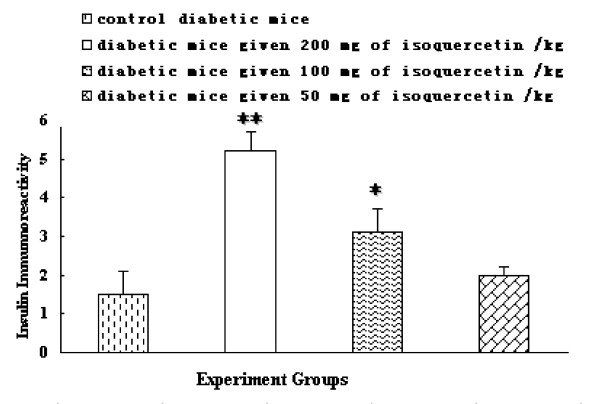
**Comparative evaluation of the expression of insulin immunoreactivity in the pancreatic islets**. **p < 0.01 and *p < 0.01 compared with the control diabetic mice.

Isoquercetin treatment group (200 mg/kg) increased the area of insulin immunoreactive β-cells in the pancreatic islets significantly compared with the diabetic group (Figure 3 and 4), suggesting that the rate of apoptotic in the islet β-cells was slowed. Numerous documents have demonstrated that oxidative stress usually promotes the production of reactive nitrogen species and reactive oxygen species, which can change in the mitochondrial membrane potential, and the release of cytochrome c, triggering cell apoptosis [29]. In view of a report by Silva [12] who reported isoquercetin play protective roles against oxidative stress, therefore isoquercetin may also protect the pancreatic β-cells from type 2 diabetes mellitus related apoptosis.

## Conclusions

Isoquercetin had a regulative role in blood glucose level and lipids, and improved the function of pancreatic islets. Isoquercetin may be useful in the treatment of type 2 diabetes mellitus.

## Competing interests

The authors declare that they have no competing interests.

## Authors' contributions

RZ conducted most of the animal experiments. YY collected and interpreted the data and wrote the manuscript. YW prepared the isoquercetin. GR designed all the experiments. All authors have read and approved the final manuscript.
